# 

**Published:** 1906-04

**Authors:** 


### MISCELLANY.

---

The United States Civil Service Commission announces an examination on June 6-7, 1906, at the usual places, to secure eligibles from which to make certification to fill at least two vacancies, at $600 per annum each, with maintainence, in the position of medical interne, Government Hospital for the Insane, Washington, D. C., and vacancies as they may occur in any branch of the service requiring similar qualifications.

The Department states that it reserves the right to continue or terminate appointment at the end of one year, or to promote the appointee at the expiration of that length of service.

